# Supplementary material for: Splice-Junction-Based Mapping of Alternative Isoforms in the Human Proteome
Source: Cell Rep. Author manuscript; Available in PMC 2020 Jan 15. (PMC6961840; doi:10.1016/j.celrep.2019.11.026)
Supplement: 2 [file NIHMS1546469-supplement-2.zip › DF1/PXD006675/RightVentricle/RightVentricle_54_RBBP4_GPDAVEERVINEEYK.pdf]

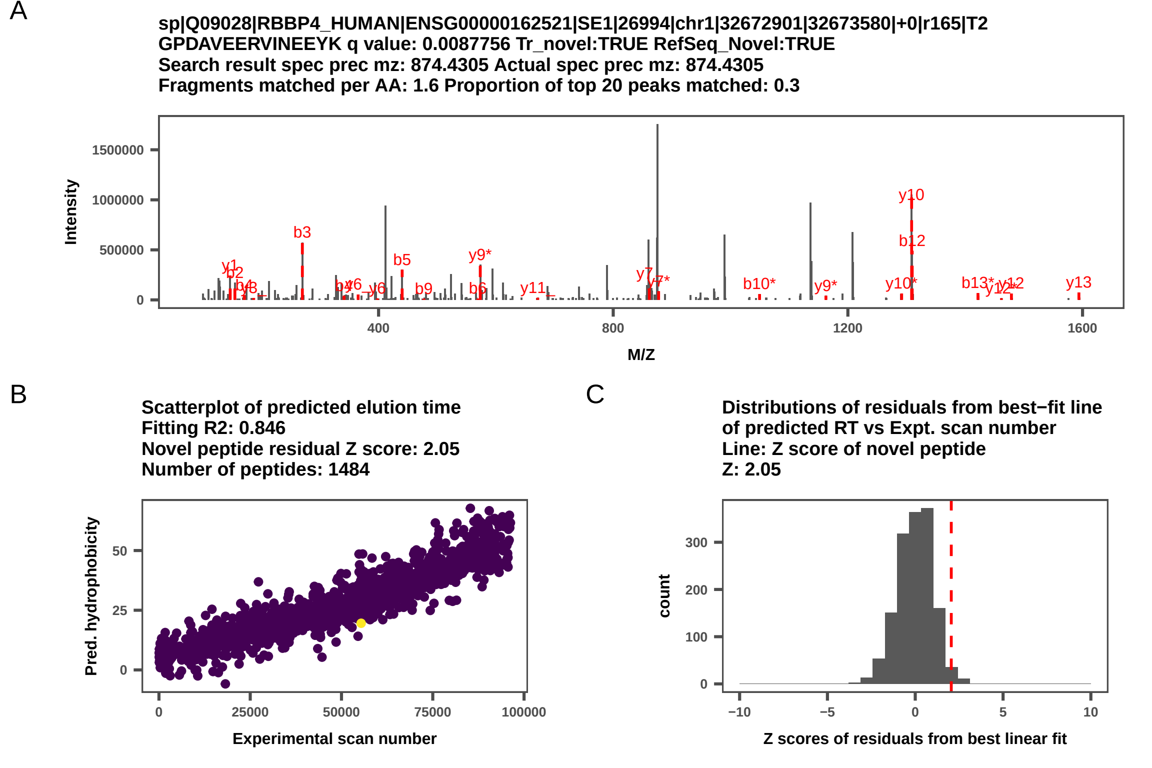
A
sp|Q09028|RBBP4_HUMAN|ENSG00000162521|SE1|26994|chr1|32672901|32673580|+0|r165|T2
GPDAVEERVINEEYK q value: 0.0087756 Tr_novel:TRUE RefSeq_Novel:TRUE
Search result spec prec mz: 874.4305 Actual spec prec mz: 874.4305
Fragments matched per AA: 1.6 Proportion of top 20 peaks matched: 0.3
Intensity
1500000
1000000
500000
0
M/Z
400
800
1200
1600
y1
b2
b3
b5
y9*
b9
b6
y11
y7
y7*
b10*
y9*
y10
b12
y10*
b13*
y12
y12*
y13
B
Scatterplot of predicted elution time
Fitting R2: 0.846
Novel peptide residual Z score: 2.05
Number of peptides: 1484
Pred. hydrophobicity
50
25
0
0
25000
50000
75000
100000
Experimental scan number
C
Distributions of residuals from best−fit line
of predicted RT vs Expt. scan number
Line: Z score of novel peptide
Z: 2.05
count
300
200
100
0
−10
−5
0
5
10
Z scores of residuals from best linear fit
